# Two-dimensional transesophageal echocardiography for aortic annular sizing in patients undergoing transcatheter aortic valve implantation

**DOI:** 10.1186/s12872-015-0181-3

**Published:** 2015-12-29

**Authors:** Mohammad A. Sherif, Hüseyin Ince, Octavian Maniuc, Therese Reiter, Wolfram Voelker, Georg Ertl, Alper Öner

**Affiliations:** Internal Medicine Centre, Cardiology Department, Rostock University Clinic, Ernst-Hyedemann- Street 6, 18057 Rostock, Germany; I. Internal Medicine Clinic, Cardiology Department, Wuerzburg University Clinic, Wuerzburg, Germany

**Keywords:** TAVI, Sizing, Echocardiography

## Abstract

**Background:**

Accurate preoperative assessment of the aortic annulus dimension is crucial for successful transcatheter aortic valve implantation (TAVI). In this study we examined the accuracy of a novel method using two-dimensional transesophageal echocardiography (2D-TEE) for measurement of the aortic annulus.

**Methods:**

We evaluated the theoretical impact of the measurement of the annulus diameter and area using the circumcircle of a triangle method on the decision to perform the procedure and choice of the prosthesis size.

**Results:**

Sixty-three consecutive patients were scheduled for TAVI. Mean age was 82 ± 4 years, and 25 patients (55.6 %) were female. Mean aortic annulus diameter was 20.3 ± 2.2 mm assessed by TEE on the mid-esophageal long-axis view and 23.9 ± 2.3 mm using CT (*p* < 0.001). There was a tendency for the TEE derived areas using the new method to be higher (*p* < 0.001). The TEE measurements were on average 42.33 mm^2^ higher than the CT measurements without an evidence of a systematic over- or under-sizing (*p* = 1.00). Agreement between TEE and CT chosen valve sizes was good overall (kappa = 0.67 and weighted kappa = 0.71). For patients who turned out to have no AR, the two methods agreed in 84.6 % of patients.

**Conclusions:**

CT remanis the gold standard in sizing of the aortic valve annulus. Nevertheless, sizing of the aortic valve annulus using TEE derived area may be helpful. The impact of integration of this method in the algorithm of aortic annulus sizing on the outcome of patients undergoing TAVI should be examined in future studies.

## Background

Accurate preoperative assessment of the aortic annulus dimension is crucial for successful transcatheter aortic valve implantation (TAVI). Two-dimensional transesophageal echocardiography (2D-TEE) may underestimates the annulus [1]. For this reason, alternative sizing methods based on multidetector computed tomography (CT) [2] und Three-dimensional (3D) TEE [3] have been developed.

CT has become the “gold standard” for non-invasive preoperative evaluation of the aortic root and aortic annulus prior to TAVI using the balloon expandable Edwards-Sapien (ES) bioprosthesis (Edwards Sapien/Sapien XT, Edwards Lifesciences, Irvine, California) [4–6].

However, the proper identification and alignment of the plane on which the virtual ring is situated might be difficult because of heavy calcifications or extremely oval annuli. Both of these issues could lead to distortion of the aortic root. In these cases, patients would benefit most from multimodality imaging [1].

In this study we examined the accuracy of a new method using 2D-TEE for non-invasive preoperative evaluation of the aortic annulus prior to TAVI using the balloon expandable ES bioprosthesis.

## Methods

### Study design and patients

The new method to measure the aortic valve annulus was applied in 63 consecutive patients with severe symptomatic tricuspid aortic stenosis (aortic valve area [AVA] <1 cm^2^ or indexed AVA <0.6 cm^2^/m^2^) were recruited and underwent successful TAVI using the balloon expandable Edwards-Sapien (ES) bioprosthesis (Edwards Sapien/Sapien XT, Edwards Lifesciences, Irvine, California through the transfemoral route.

Accordingly, a theoretical decision was made for the size of the valve to be implanted; the virtual valve. The echocardiographers were blinded regarding the name of the patients and the measurements of the CT. These patients have undergone TAVI; nevertheless, the annulus measurement used for the procedure was done using CT as a standard in our institution. Manual reconstructions were performed with clinical software (Siemens Syngo Dynamics VIA; Siemens Healthcare, Erlangen, Germany) in a standard fashion [7]. Briefly, using the 3D multiplanar reformation (MPR) tool, the analysis plane was shifted and rotated to intersect the 3 lowest insertion points of the aortic valve leaflets. At this level, planimetry of the annulus lumen was performed. Accordingly, the area of the annulus and the mean diameter were calculated. The valve size was chosen according to the valve-sizing chart recommended from the manufacturer.

TAVI was done using the balloon expandable ES bioprosthesis. Post-operatively, the presence, degree and type (paravalvular versus transvalvular) of aortic regurgitation (AR) were recorded in all patients using TTE and aortography and quantified according to the VARC criteria [8].

Because of the nature of the study, an ethical approval was not required for this study.

### Echocardiographic assessment

A comprehensive TTE and TEE was performed preoperatively. The severity of aortic stenosis was assessed by the transvalvular mean gradient and aortic valve area (AVA), which was calculated with the continuity equation and planimetry [9].

### Circumcircle of a triangle method

**Fig. 1:**
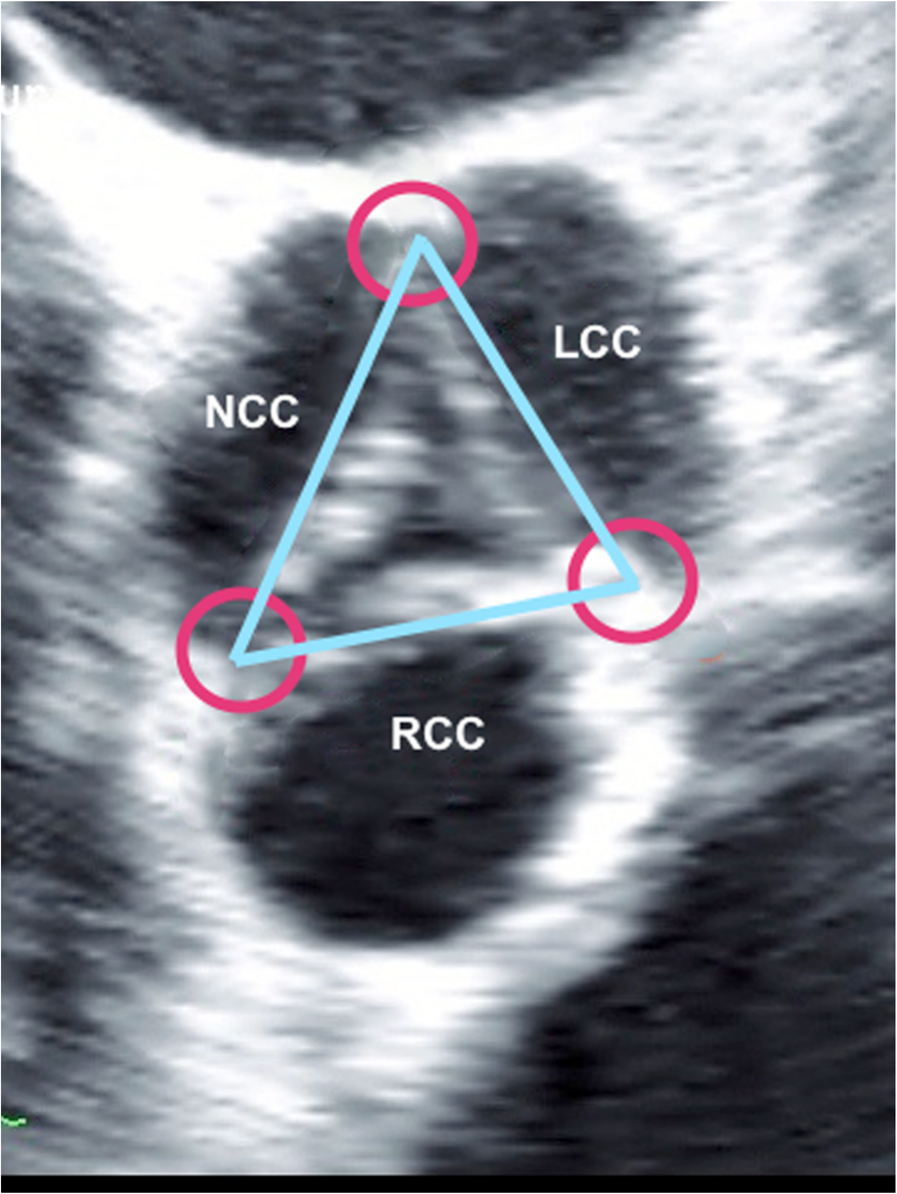
Annular size measurement using the enlarged view of the mid-oesophageal short axis (approximately 30° to 50°). The short-axis views of the aortic valve are generated at the insertion of the cusps in early systole. Red circles identify the thickened parts at the sites of peripheral attachment of the zones of apposition between the aortic valve leaflets. They represent also the vertices of the interleaflet triangles. LCC: left coronary cusp, RCC: right coronary cusp, NCC: non-coronary cusp

**Fig. 2:**
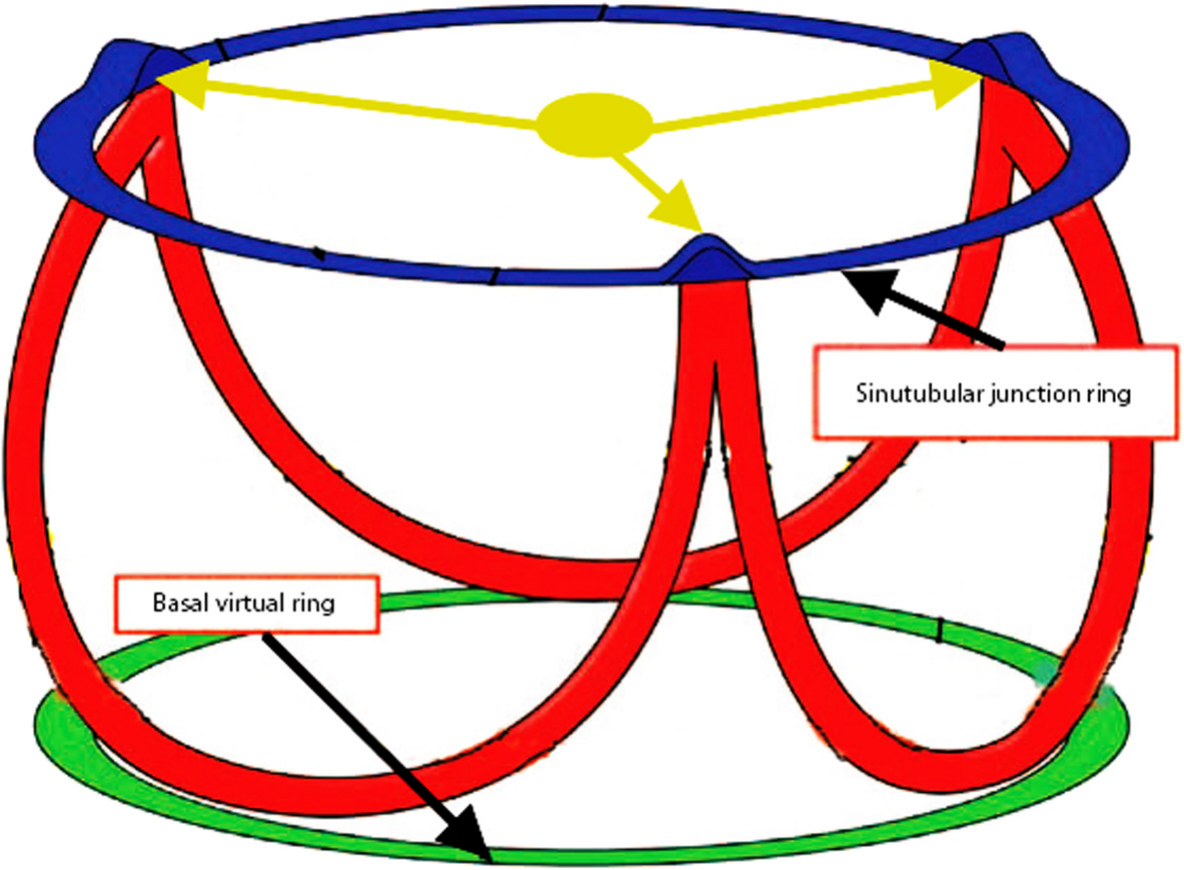
Schematic drawing describes the anatomic arrangement of the aortic valve leaflets supported in crown-like fashion. The diameter at the level of the basal attachment of the leaflets (*green* ring) is the one used for sizing using CT. The top of the crown, namely the sinutubular junction is the level used to measure the annulus in the study (*blue* ring)

**Fig. 3:**
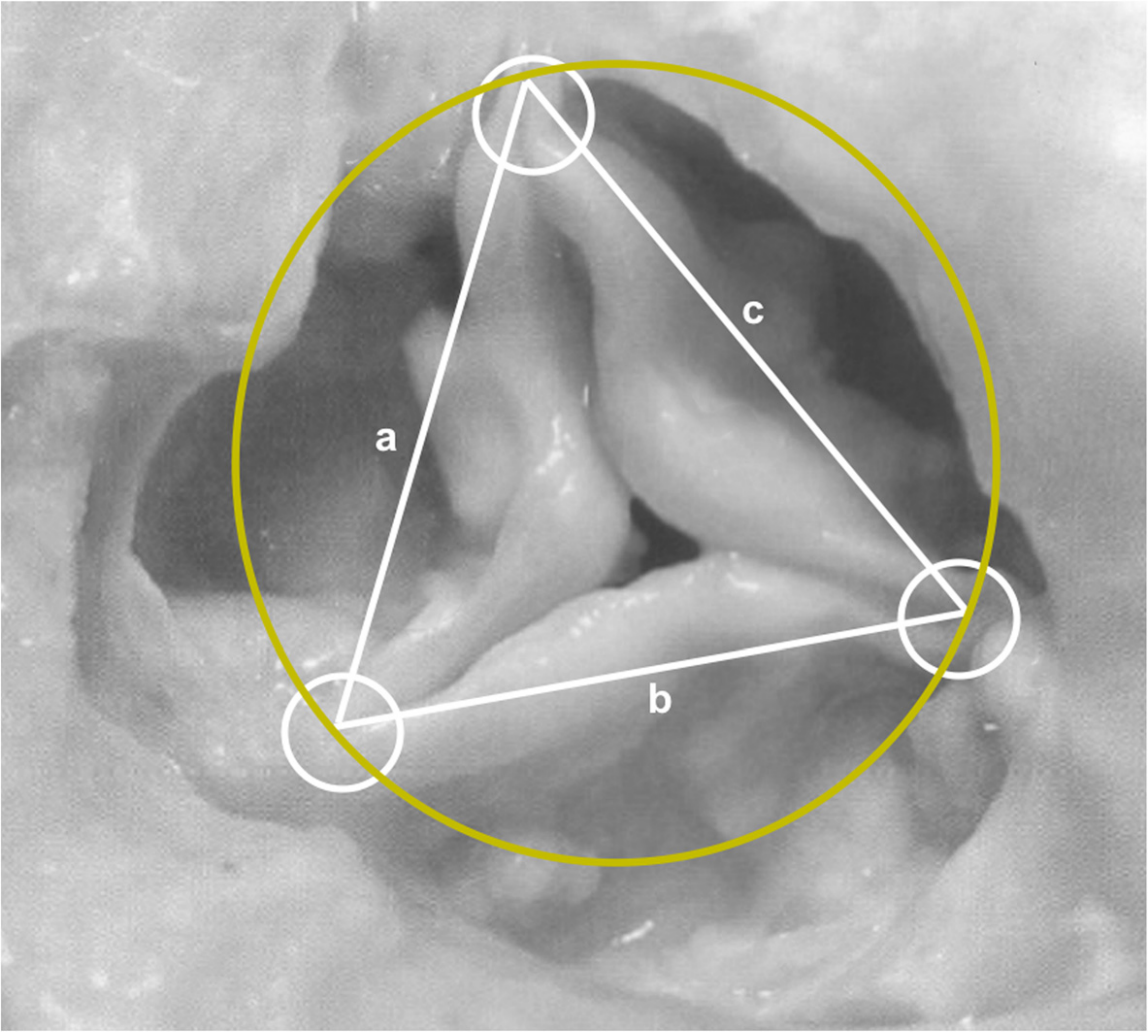
Schematic drawing describes the circumcircle of a triangle method. Three points are defined (*white* circles). Three lines are drawn between these points (a,b,c) and the resulting triangle issued to identify the circle (*yellow* circle) that intersects the three vertices of the triangle

Annular size measurement was performed using the enlarged view of the mid-esophageal short axis (approximately 30° to 50°) during the early systolic phase of the cardiac cycle. The short-axis views of the aortic valve were generated at the insertion of the cusps in systole. The mid-esophageal AV short axis view was obtained from the mid-esophageal window by advancing or withdrawing the probe until the AV comes into view and then turning the probe to center the AV in the display. The image depth was adjusted to between 10 to 12 cm to position the AV in the middle of the display. Next, the multiplane angle was rotated forward to approximately 30 to 60° until a symmetrical image of all three cusps of the aortic valve and the coronary sinuses comes into view. This view shows how the leaflets join together along trifoliate zones of apposition extending from peripheral attachments at the sinutubular junction to the centroid of the valvular orifice (Fig. 1). These zones of apposition are the true commissures. The aortal end of the commissures correlates anatomically to the upper end of the inter-leaflet triangle and at the same time they represent the sinutubular junction (blue ring in Fig. 2). Three points were defined (red circles Fig. 1). Three lines were drawn between these points and the resulting triangle was used to identify the circle that intersects the three vertices of the triangle i.e. circumcircle of the triangle (yellow circle in Fig. 3).

The area and the radius of this circle were calculated und accordingly the size of the valve was chosen.

Knowing the length (a,b,c) of the three sides of the triangle (Fig. 3), the radius of its circumcircle was calculated.

### Statistical analysis

Statistical analysis was done using Minitab software (Minitab, Release 13.1, State College, Pennsylvania). Data were expressed as mean ± SD or percent. Comparisons of measurements and valve sizes according to the methodology were performed using the *t*-test or chi-square test as appropriate.

The Bland-Altman analysis was done to see whether there is any bias between the different methods of sizing, i.e. whether the mean values for the methods differ. Two standard measures of agreement between the valve sizes for each method were also done: kappa and weighted kappa. As a rough guide, values of kappa less than or equal to 0.2 suggest poor agreement, values between 0.2 and 0.40 suggest fair agreement, values between 0.4 and 0.6 suggest moderate agreement, values between 0.6 and 0.8 suggest good agreement and values between 0.8 and 1.0 suggest very good agreement. The difference between kappa and weighted kappa, is that weighted kappa takes into account that a valve size of 23 by CT and 29 by TEE suggests worse agreement than, a sizing of 23 by CT and 26 by TEE. The supporting data are available if required.

## Results

Sixty-three consecutive patients were scheduled for TAVI received TTE, TEE, and CT in our hospital. Mean age was 82 ± 4 years, and 35 patients (55.6 %) were female. Atrial fibrillation was present in 32 patients (50.8 %). All patients had an anatomical tricuspid aortic valve. Mean aortic valve area was 0.74 ± 0.31 cm^2^, and mean gradient was 47.5 ± 14.6 mmHg.

Mean aortic annulus diameter was 20.3 ± 2.2 mm assessed by TEE on the mid-esophageal long-axis view and 23.9 ± 2.3 mm using CT (*p* < 0.001) (Table 1).

**Table 1:** Clinical, echocardiographic and procedural characteristics of the study population

Variable	Mean ± SD
Age, years	82.4 ± 5.5
Female gender, %	55.6
Atrial fibrillation, %	50.8
Ejection fraction, %	55 ± 10
Aortic valve area, cm^2^	0.74 ± 0.31
Peak pressure gradient, mmHg	72.6 ± 21.4
Mean pressure gradient, mmHg	47.5 ± 14.6
Annulus diameter using TEE hinge-to-hinge (long axis view), mm	20.3 ± 2.2
Annulus diameter using CT, mm	23.9 ± 2.3
Annulus diameter according to circumcircle of a triangle method, mm	25.6 ± 2.4
Annulus area using CT, mm^2^	453.3 ± 77.7
Annulus area according to circumcircle of a triangle method, mm^2^	495.6 ± 95.7
Access site	
Femoral	65 %
Apical	35 %
Valve size	
23-mm	36.5 %
26-mm	47.6 %
29-mm	15.9 %
Post-operative AR (angiographic)	
No	62 %
Minimal/mild	30 %
Moderate	8 %
Post-operative AR (echocardiographic)	
No	51 %
Minimal/mild	38 %
Moderate	11 %
Post-operative peak gradient, mmHg	20.6 ± 6.5
Post-operative mean gradient, mmHg	11.8 ± 3.8

### Theoretical impact of the method of measurement of the annulus on the procedure

We evaluated the theoretical impact of the measurement of the annulus diameter and area assessed by TEE on the decision to perform the procedure and choice of the prosthesis size.

We hypothesized the sizing using CT as the ‘gold standard’ and compared the choice of the ES valve based on it to the choice based on TEE.

### Comparing valve selection by CT and TEE derived area

**Fig. 4:**
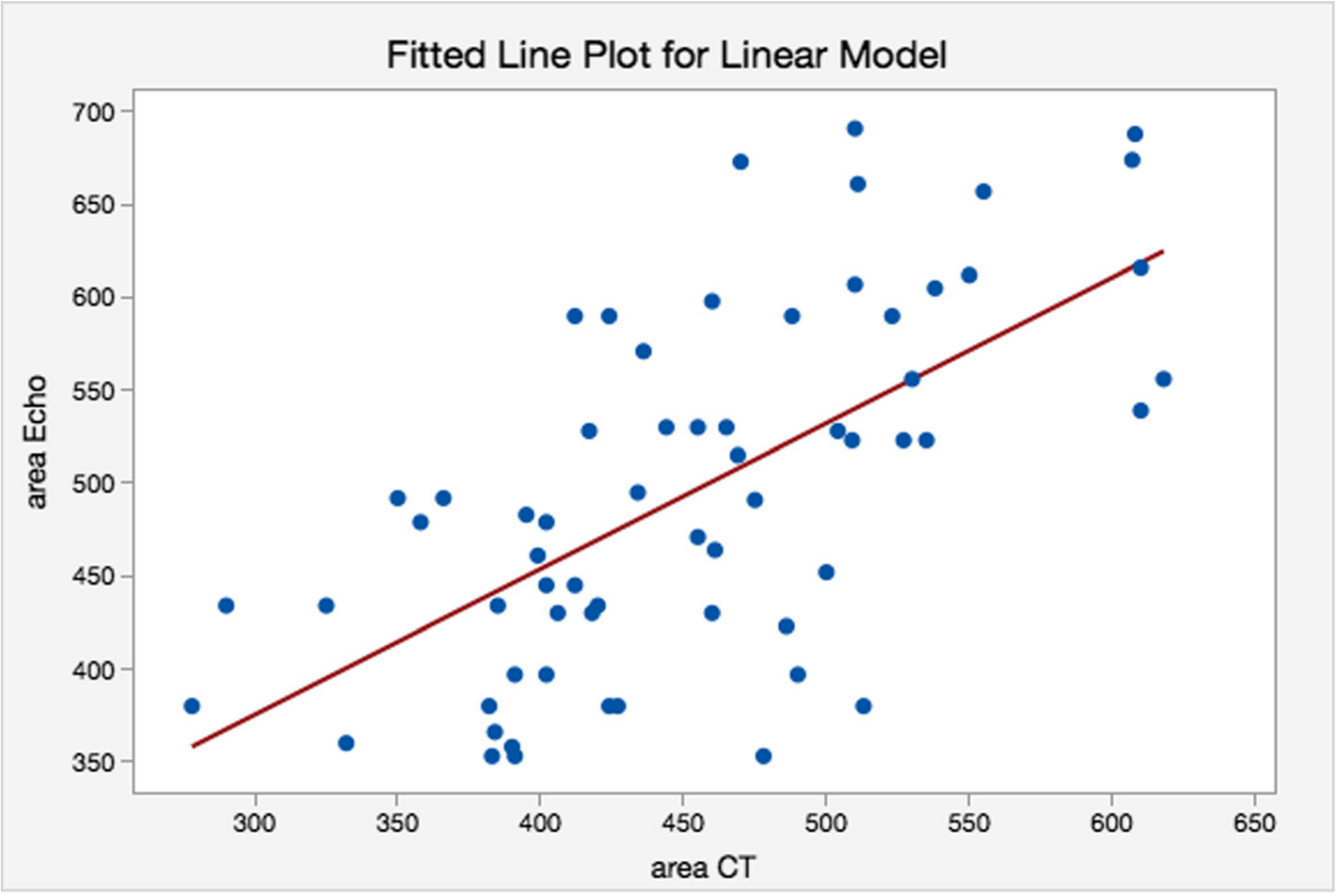
A scatter plot of TEE derived area against CT derived area. The equation of the regression line is: area Echo = 140 + 0.785 area/CT

**Fig. 5:**
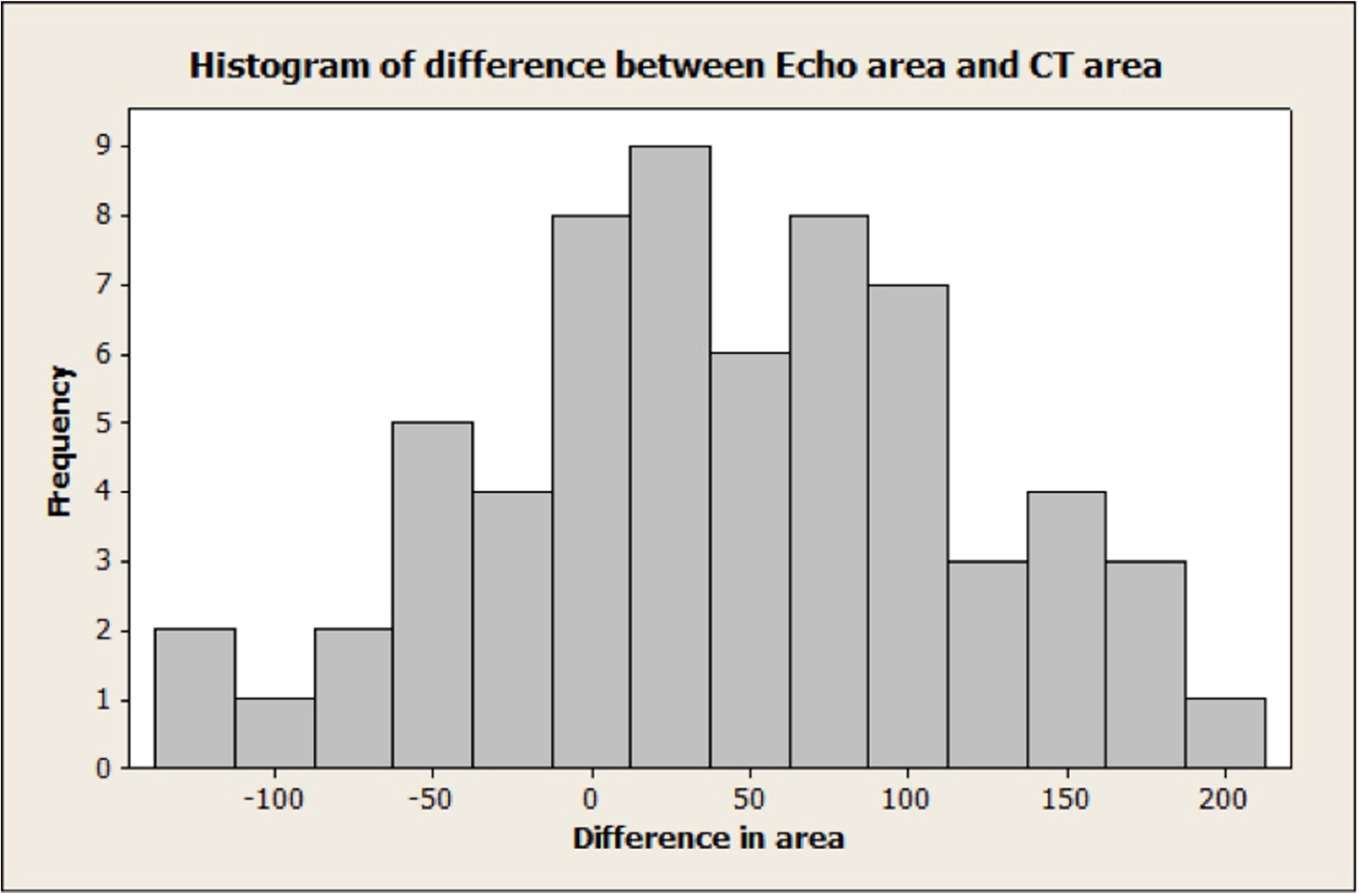
A histogram of the difference: TEE derived area minus CT derived area. There is a tendency for the echo areas to be higher (*p* < 0.001, *t*-test)

A Comparison of areas as estimated using CT and TEE and the difference between them is shown in Figs. 4 and 5. There was a tendency for the TEE derived areas to be higher (*p* < 0.001, *t*-test). Interestingly, the mean difference was only 28.5 mm^2^ for patients who subsequently had no AR and was 64.8 mm^2^ for patients who had AR grade 1 or 2, but this difference was not statistically significant (*p* = 0.075, two-sample *t*-test).

The Bland-Altman analysis showed that TEE measurements are on average 42.33 mm^2^ higher than the CT measurements and that this difference is statistically significant (*p* < 0.001).

**Fig. 6:**
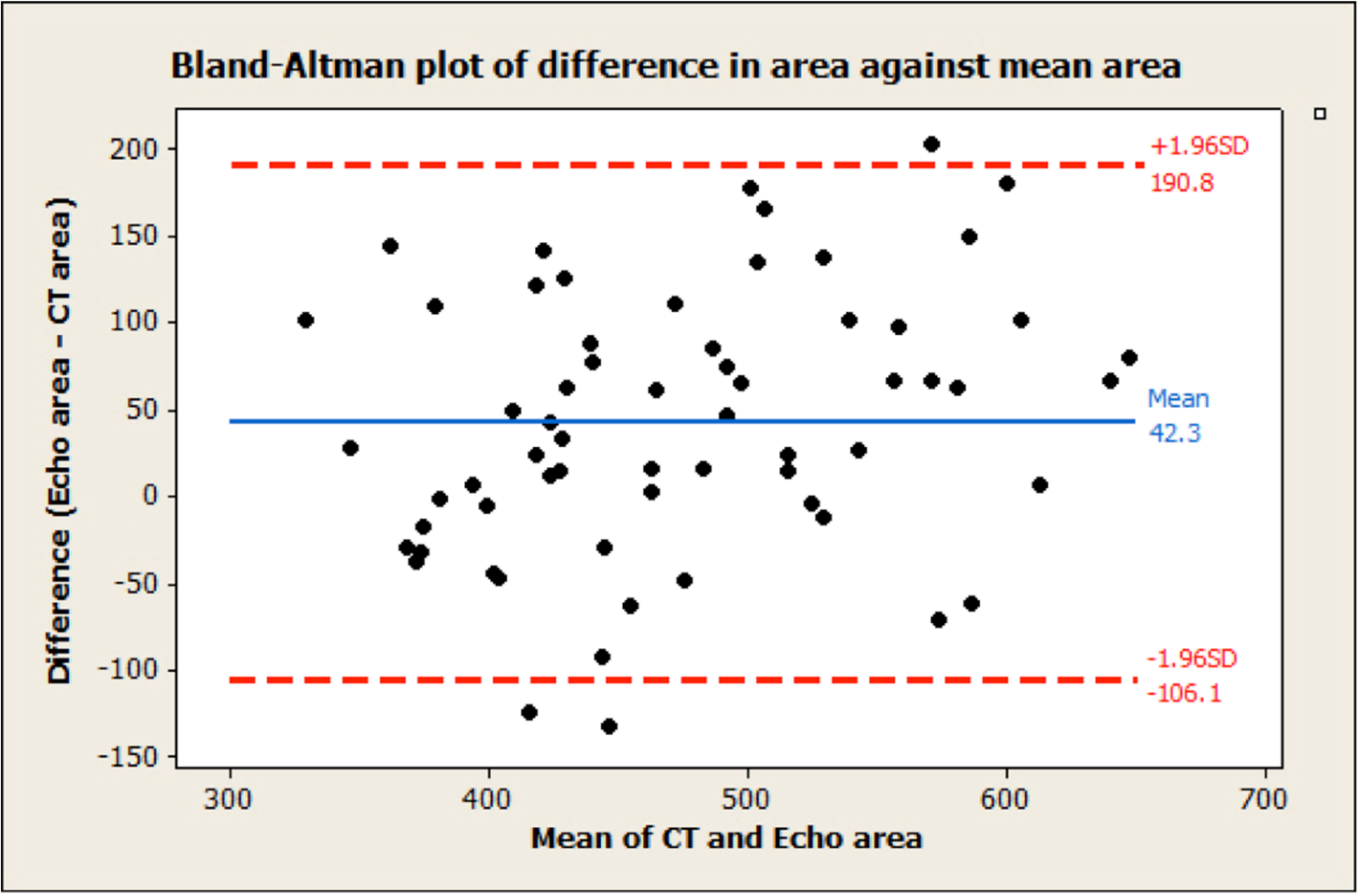
The Bland-Altman plot of the difference (TEE derived area minus CT derived area) against the mean area of TEE and CT. The red dotted lines show the range of differences between the measurements for most (about 95 %) of individuals

The Bland-Altman plot of the difference (TEE derived area minus CT derived area) against the mean area by TEE and CT is shown in Fig. 6. The red dotted lines show the range of differences between the measurements for most (about 95 %) of individuals.

**Fig. 7:**
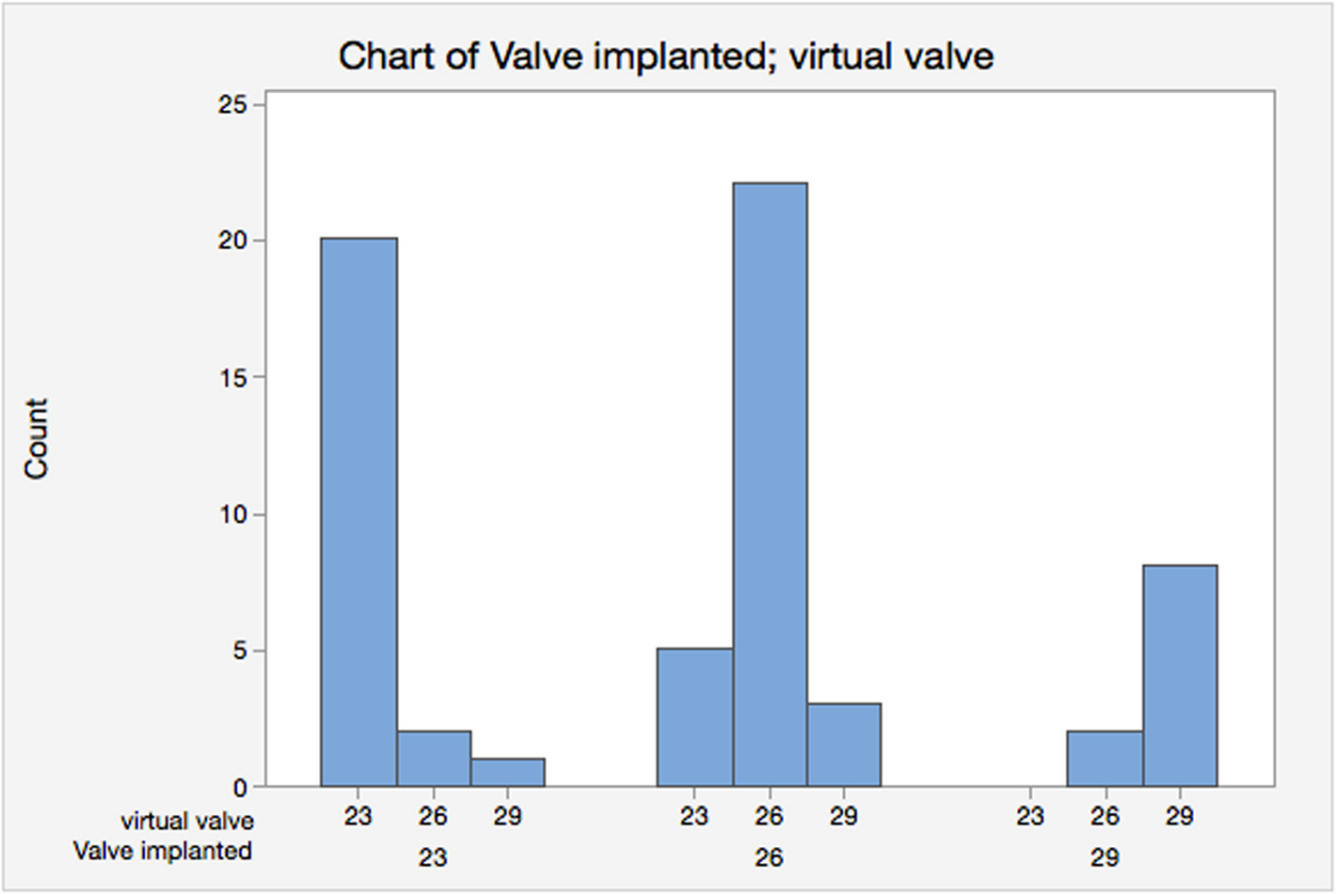
Shows the frequencies of the virtual valve sizes according to TEE derived area in comparison to the real valve sizes

Figure 7 shows the frequencies of the virtual valve sizes according to TEE derived area in comparison to the real valve sizes. We see that the same sizing was made in 50 out of the 63 patients (79.5 %); valve sizes using TEE were higher for 6 patients (9.5 %) and lower for 7 patients (11 %). There does not seem to be much evidence of a systematic over- or under-sizing (*p* = 1.00). Agreement between TEE and CT chosen valve sizes overall was good (kappa = 0.67 and weighted kappa = 0.71).

**Fig. 8:**
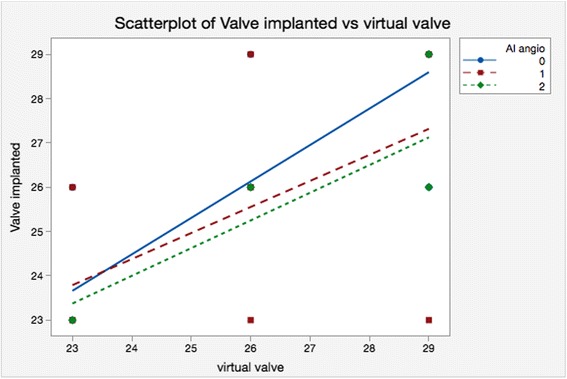
Scatterplot of the implanted valve versus the virtual valve according to the degree of the AR

For patients who turned out to have no AR, the two methods agreed in 84.6 % of patients; for patients who turned out to have AR grade 1, the two methods agreed in 73.7 % of patients; for patients who turned out to have AR grade 2, the two methods agreed in 60.0 % of patients. So it could be argued that the TEE derived area method disagreed with the CT method more often when the sizing using CT turned out to be unsatisfactory (Fig. 8).

However, this conclusion is based on very low frequencies and so is almost certainly not statistically significant.

## Discussion

We analyzed the influence of different measurement techniques on the theoretical valve size selection in our patient population. Valve choice based on the TEE derived area demonstrated the best agreement to valve choice based on CT. The disagreement was more pronounced in the patients who had any degree of post-operative AR.

The indications for transcatheter prosthesis size selection provided by the manufacturers were based on 2D-TEE measurements [10]. This technique was used to accurately determine the size of the aortic annulus and the excellent results of TAVI reported to date may be attributable to these complementary techniques [1, 11–13].

The main limitation of echocardiography relates to its two-dimensional nature. The aortic root in fact has a complex three-dimensional geometry and the semi-lunar attachments of the aortic cusps take the shape of a 3-pronged coronet. Working on a uni-planar view leads to the possibility of underestimating or overestimating the annular size, due to the fact that the actual plane of the section may lie out of the annulus center [14].

3-D TEE and a multi-planar reconstruction of the aortic root and of the outflow tract can overcome this limitation but the capacity to visualize the entire annulus can be limited by the calcifications [15].

An oversized prosthetic valve relative to the dimensions of the patient’s aortic root can result in redundancy of leaflet tissue, thus creating folds. These folds will generate regions of compressive and tensile stresses and may alter the function or reduce the durability of the valve [16]. On the other hand, if the prosthesis is too small for the patient, the incidence of significant paravalvular regurgitation is high.

Anderson et al. [17] mentioned that, the essence of the anatomic arrangement of the aortic root is that the aortic valve leaflets are supported in crown-like fashion within the cylindrical root.

The diameter at the level of the basal attachment of the leaflets (green ring in Fig. 2) is the diameter that is usually defined as the valvular annulus. Nevertheless, it is no more than a virtual ring and does not correspond with the annulus defined by cardiac surgeons [17]. This level is the one used for sizing using CT.

In this study we used the top of the crown, which is a true anatomical ring, namely the sinutubular junction as a surrogate for the aortic valve annulus (blue ring in Fig. 2). It is thickened at the sites of peripheral attachment of the zones of apposition between the aortic valve leaflets (Red circles in Fig. 1).

In general, the diameter at the level of the sinutubular junction exceeds that at the level of the virtual basal ring by up to one-fifth [18]. This agrees with our findings, as we found that using the diameter for sizing was associated with a tendency to oversizing.

Moreover, the aortic root is a dynamic structure, with its geometric parameters changing continuously both during the phases of the cardiac cycle and in relation to changes in pressure within the aortic root [19]. From diastole to systole, the diameter at the level of the outlet has been noted to increase by 12 %, while the diameter at the base decreases by 16 % [20].

CT has become the “gold standard” for measuring the aortic valve annulus as it allows a detailed understanding of the complex three-dimensional aortic root anatomy, including the crown-shaped anatomic aortic annulus, the virtual basal ring, and the sinuses of Valsalva with the origin of the coronary arteries.

Several studies [5, 21] have found that aortic annular sizing using CT in patients undergoing TAVI using the balloon expandable ES prosthesis is the most accurate method. Nevertheless, radiation exposure, iodine injection and costs are important limitations in comparison to using TEE. Moreover, over 50 % of patients undergoing TAVI in large studies had pre-existing chronic kidney disease and about 10 % of these patients have severe renal insufficiency [22, 23].

It should be observed, however, that the exact identification of the plane on which the basal ring lays is not always straightforward in clinical practice. In fact, the nadir of any particular cusp is located at the point where that cusp is “seen to disappear,” and the operator judges that the plane is correctly oriented when the three cusps disappear all together, an occurrence that may be hard to reproduce in some patients, such as those with heavy or asymmetrical annular calcifications or an extremely elliptical annulus. While it is easy to identify the “height” of the nadir of a cusp in the aortic root on the sagittal and coronal oblique views, its angular position on the annular circumference may be hard to locate in some patients [15].

Several scan protocols for TAVI assessment have been developed. The technical details on how to obtain a good scan are beyond the scope of this article, but a high quality acquisition without artifacts is a pre-requisite for all the post-processing and image analysis [24].

It has recently been demonstrated that TAVI alters the geometry of the aortic annulus to a more circular configuration [15, 25]. Therefore, we think that using the TEE derived area according to the circumcircle of a triangle method may accurately predicts the size of the valve as the area generated mimics the area of the bioprosthesis to be implanted.

Most importantly, the present study demonstrates that the TEE derived area may be a plausible method for measuring the aortic annulus and provide accurate information comparable to CT.

For borderline aortic annulus dimensions, ES size selection may be challenging. This TEE method may help the operator with an extra tool to validate, if not to correct, the measurements done using CT.

## Conclusions

CT remanis the gold standard in sizing of the aortic valve annulus. Nevertheless, sizing of the aortic valve annulus using TEE derived area may give additional information. The impact of integration of this method in the algorithm of aortic annulus sizing on the outcome of patients undergoing TAVI should be examined in future studies.

### Limitations

This study is a non-randomized study having its inherent limitations. A randomized non-inferiority or better superiority study comparing both methods and their impact on the outcome regarding the incidence of postoperative AR, aortic annulus rupture and valve embolization is warranted. The small number of patients is a limitation in this study, but it is the first step to validate this novel method. All patients received an ES balloon-expandable prosthesis. Therefore, findings of this study may not apply to the self-expandable prosthesis.

## Abbreviations

AR: Aortic regurgitation
AV: Aortic valve
AVA: Aortic valve area
CT: Multidetector computed tomography
ES: Edwards-Sapien
LVOT: Left ventricular outflow tract
LCC: Left coronary cusp
MPR: Multiplanar reformation
NCC: Non-coronary cusp
RCC: Right coronary cusp
TAVI: Transcatheter aortic valve implantation
TTE: Transthoracic echocardiography
2D-TEE: Two-dimensional transesophageal echocardiography
3D-TEE: Three-dimensional transesophageal echocardiography
